# Prediction of Sleep Quality in Cancer Survivors Based on Arousal, Pain, and Worry: The Mediating Role of Dysfunctional Beliefs and Attitudes About Sleep

**DOI:** 10.1002/cam4.70773

**Published:** 2025-04-01

**Authors:** Omid Amani, Mohammad Ali Mazaheri, Mona Malekzadeh Moghani, Fariba Zarani

**Affiliations:** ^1^ Department of Psychology Shahid Beheshti University Tehran Iran; ^2^ Department of Radiation Oncology Shahid Beheshti University of Medical Sciences Tehran Iran

**Keywords:** arousal, cancer, pain, sleep quality, worry

## Abstract

**Purpose:**

Our study explores the prevalence, severity, and psychological correlates of insomnia in cancer survivors, aiming to predict sleep quality based on pre‐sleep arousal, pain, and worry, while examining the mediating role of dysfunctional beliefs and attitudes about sleep (DBAS).

**Methods:**

A descriptive‐correlational design was employed, with 200 cancer survivors from Tehran, Iran, selected through convenience sampling in 2022. Participants completed the Pittsburgh Sleep Quality Index (PSQI), Chronic Pain Grade (CPG), Dysfunctional Beliefs and Attitudes about Sleep Scale (DBAS), Pennsylvania Worry Questionnaire (PSWQ), and Pre‐Sleep Arousal Scale (PSAS). The data were analyzed using correlation and multiple regression through SPSS‐23 software.

**Results:**

On average, 37.07 months post‐treatment, 95% of survivors reported delayed sleep onset, 88.5% frequent awakenings, 72% reduced sleep duration, and 67% morning dysfunction. Significant positive associations were found between pre‐sleep arousal, chronic pain (*r* = 0.552), worry (*r* = 0.161), DBAS (*r* = 0.363), and poor sleep quality (*r* = 0.607). Regression analysis indicated that physical arousal (*B* = 0.29, *p* = 0.01) and DBAS (*B* = 0.14, *p* < 0.05) were significant predictors of sleep quality, with DBAS mediating the relationship between physical arousal and sleep quality.

**Conclusions:**

Persistent sleep problems after cancer treatment highlight the need for targeted interventions in survivorship care. Sleep‐focused strategies may improve sleep quality and reduce the burden of insomnia‐related issues.

**Implications for Cancer Survivors:**

Addressing pre‐sleep arousal, pain, worry, and DBAS through targeted interventions is crucial for improving sleep quality and overall quality of life in cancer survivors.

## 1 Introduction

Cancer is a chronic disease that affects people of all ages, genders, and races, with a profound impact on both individual and public health [1, 2]. It is the second leading cause of death in developed countries and ranks third in developing countries like Iran, following accidents and cardiovascular diseases [3]. Among the challenges faced by cancer survivors is the high prevalence of sleep problems, which can significantly affect their quality of life. These sleep disturbances are often influenced by multiple factors, including physical and psychological variables, highlighting the need for comprehensive research in this area. As identified in a systematic review, 1 to 2 years after the last course of radiotherapy and chemotherapy, survivors report multiple issues such as fatigue (16%–49%), anxiety (45%–48%), and pain (26%–47%), which directly and indirectly contribute to sleep problems (59%) in cancer survivors [4]. The results of other studies also indicated the persistence of sleep problems for 2 years after the completion of treatment [5]. A study revealed that 63 months after treatment, 38% of cancer survivors experienced poor sleep quality, required more time to fall asleep, woke up more frequently, and, on average, slept 2 h less than their peers. This was strongly associated with higher pain levels, greater concerns about health and disease recurrence, and ultimately a lower quality of life for the affected individuals [6].

Research consistently shows that sleep problems are highly prevalent across all types of cancer, with rates ranging from 59% to 79% [5, 7, 8], and most cancer survivors have reported that poor sleep quality (the degree to which an individual perceives their sleep to be restful and restorative) and difficulty staying asleep throughout the night are among their greatest challenges [9]. These problems are accompanied by disruptions in the individual's daily functioning, including fatigue, frequent napping, low energy, and reduced activity levels [10]. Alongside the assessment of the prevalence of this disorder, a set of physical and psychological variables affecting the sleep disturbances (any disruptions or difficulties in sleep patterns) of cancer survivors has been identified. By evaluating the role of these factors and aiming to reduce them, the severity of insomnia in this group can be reduced. For instance, research has shown that cancer survivors who struggle with sleep are often burdened by distressing and stressful thoughts, with pain, worry, anxiety, and negative thinking emerging as key symptoms in those suffering from insomnia [11]. Chemotherapy has been shown to cause sensory nerve degeneration, leading to neuropathic pain [12]. Most cancer survivors experience late and long‐term effects, including pain, fatigue, and psychosocial challenges such as anxiety, as well as difficulties in social activities, work, and family responsibilities [13, 14]. Sleep efficiency (the proportion of time in bed that a person is asleep) can be influenced by pre‐sleep arousal (the level of cognitive and physiological activation a person experiences before bed) [15].

Morin [16], in his integrated model, considers insomnia to result from the interaction between dysfunctional beliefs and cognitions, maladaptive habits and behaviors, worry about the consequences of sleep deprivation, and the development of issues such as fatigue, reduced performance, and lowered mood, arousal and its dimensions, including physiological, cognitive, and emotional arousal. Cancer survivors are often worried about recurrence and metastasis, and most of them, during the post‐treatment phase, experience issues such as hot flashes, night sweats, sleep problems like insomnia, weight gain, joint stiffness, and concerns related to worry, stress, anxiety, and multiple side effects [17]. According to this model, hyperarousal plays a mediating role in insomnia, and since arousal regulates the balance between sleep and wakefulness, a high level of arousal is incompatible with sleep and can manifest in verbal (emotional‐cognitive), behavioral, and physiological ways (through the autonomic and central nervous systems). This model suggests that a set of stimulating conditions can push arousal beyond its threshold and, by disrupting the sequence of relaxation, cause difficulties in initiating sleep. These stimulating conditions may also include external stressors that occur during the day and manifest as sleep deprivation. In this regard, Rumble et al. in a study on cancer survivors, found that high levels of sleep‐inhibiting thoughts and behaviors are among the precursors to insomnia, and high levels of pain, fatigue, hot flashes, and low levels of positive mood are its consequences [18].

Furthermore, Carpenter et al. reported that sleep disturbances in cancer survivors were associated with higher levels of depression and fatigue, and these factors predicted a greater percentage of sleep problems compared to hot flashes [19]. Despite this substantial body of findings, the existing evidence on which factors play a greater role in causing sleep disturbances is still unclear. A vast body of research literature indicates that untreated chronic insomnia negatively affects mood symptoms, physical symptoms, pain sensitivity, fatigue, and quality of life [20], and insomnia is increasingly being recognized as an independent risk factor for future depression [21]. Thus, conducting studies aimed at addressing this issue has the potential to improve the quality of life of cancer survivors by reducing physical symptoms and helping to improve their mood after the illness. Given that the importance of understanding sleep disorders in cancer survivors has been specifically emphasized by the U.S. Department of Health and Human Services [22], and considering that long‐term sleep problems have numerous negative psychological and physical consequences [20], the quality of sleep in cancer survivors who have lived for years after their last treatment has not been well studied. With more focused research in this area, symptom screening and management could become a standard part of supportive care for the cancer community. Our study examined the prevalence, severity, and nature of insomnia‐related complaints in cancer survivors who were 1 to 5 years post‐treatment, and this study aimed to predict sleep quality in cancer survivors based on the components of arousal, pain, and worry, as well as the mediating role of dysfunctional beliefs and attitudes about sleep.

## 2 Method

Our study is classified as fundamental in its objective and utilizes a descriptive‐correlational approach for data collection. The study's target population comprised all cancer survivors who attended the oncology center at Shohada‐e‐Tajrish Hospital in Tehran, Iran, between May 2022 and January 2023. Following Klein's guidelines, a sample size of 3 to 5 respondents per questionnaire item is considered adequate, with a minimum sample size of 200 participants deemed appropriate for robust analysis [23]. To minimize potential dropouts and enhance the study's validity, a total of 220 participants were recruited through convenience sampling. After applying the inclusion and exclusion criteria and removing outliers, the final sample size was reduced to 200 participants.

### 2.1 Inclusion and Exclusion Criteria

The inclusion criteria required participants to have a diagnosis of a single type of cancer, with at least 1 year but no more than 5 years since their completion of active cancer treatment. Participants needed to have no history of multiple cancers, no concurrent physical illness at the time of assessment, and no psychiatric disorders that could interfere with data collection. Additionally, participants had to be at least 18 years old, able to read and write, and provide written informed consent. Exclusion criteria included any diagnosed psychiatric disorders, any concurrent chronic physical illnesses, withdrawal from the study, and incomplete or unclear questionnaire responses.

### 2.2 Measures

#### 2.2.1 Demographic Characteristics Questionnaire and Clinical History Interview

A demographic questionnaire collected data on age, gender, marital status, education, employment, medical history, past surgeries, cancer diagnosis, treatment type, and time since treatment. A clinical history interview was conducted to assess eligibility.

#### 2.2.2 Pittsburgh Sleep Quality Index (PSQI)

PSQI is a self‐report tool with 18 questions assessing sleep quality over the past 4 weeks [24]. Scoring ranges indicate poor sleep quality. For further information about the PSQI, see Supporting Information S1.

#### 2.2.3 Dysfunctional Beliefs and Attitudes About Sleep Scale‐10 (DBAS‐10)

This tool assesses dysfunctional beliefs about sleep with 10 items rated on a 5‐point Likert scale [25]. Scores range from 10 to 50. For further information about the DBAS‐10 [26], see Supporting Information S1.

#### 2.2.4 Chronic Pain Grade (CPG)

This questionnaire measures chronic pain intensity [20] Respondents rate seven items on an 11‐point scale [27]. For further information about the CPG, see Supporting Information S1.

#### 2.2.5 Pennsylvania Worry Questionnaire (PSWQ)

This self‐report tool consists of 16 questions, scored on a 5‐point Likert scale, assessing worry. Total scores range from 16 to 80 [26]. For further information about the PSWQ, see Supporting Information S1.

#### 2.2.6 Pre‐Sleep Arousal Scale (PSAS)

This tool comprises 16 items that assess physical and cognitive pre‐sleep arousal. Responses are rated on a 5‐point Likert scale [28]. For further information about the PSAS, see Supporting Information S1.

### 2.3 Procedure

Our study procedure began after receiving ethical approval. Initially, the researchers visited the oncology center of Shohada‐e‐Tajrish Hospital and engaged with cancer survivors attending follow‐up appointments after completing their treatment, inviting them to participate in our study. After securing initial consent, the participants were individually invited to a quiet room, free from any environmental stimuli that might affect the process of completing the questionnaires. Participants' preliminary information was then collected, and, in adherence to ethical research principles, including confidentiality, detailed information about the study was provided to them, and informed consent was obtained. After obtaining informed consent, the participants completed the study questionnaires during a single session, with the researcher available to assist and address any questions or uncertainties.

### 2.4 Statistical Analysis

Data were analyzed using SPSS version 23. Descriptive statistics were calculated, including means and standard deviations for continuous variables and frequencies and percentages for categorical variables. Pearson's correlation coefficients were used to examine the relationships among all study variables. A series of hierarchical multiple regressions were conducted to predict the sleep quality scores. The independent variables, based on their significance to the field, were entered stepwise into the regression models. The significance level was set at *p* < 0.05. To explore the mediating role of dysfunctional beliefs and attitudes about sleep (DBAS), we conducted hierarchical regression analyses, as specified by Baron and Kenny (1986) for mediation. We assessed if: (1) the independent variable (e.g., pre‐sleep arousal) significantly predicted the mediator (DBAS); (2) the independent variable significantly predicted the dependent variable (sleep quality); and (3) with the mediator (DBAS) included in the model, the effect of the independent variable was attenuated and the mediator significantly predicted the dependent variable. The mediation model was confirmed when all these criteria were met.

## 3 Results

The results of the demographic data analysis showed that the mean age of the participants was 52.22 years, with a standard deviation of 12.087. Among the participants, 88.5% were women and 11.5% were men. Most participants had a high school diploma (35%) and the majority were housewives (66.5%). Additionally, 93.5% of the participants were married, divorced, or widowed, while only 6.5% were single. The highest frequency of the number of children, at 42%, was found in families with two children, and 74% of the participants had a history of surgery. Please see Table 1 for detailed demographic information. The average time since diagnosis was 49.9 months, the average time since the onset of the disease was 27.15 months, the average number of chemotherapy sessions was 9, the average number of radiotherapy sessions was 21, and the average follow‐up period after the illness was 37 months (see Table 1).

**TABLE 1:** Demographic description of respondents.

Variable	Frequency (%)	*M* ± SD
Age		
Under 30 years	5 (2.5)	52.220 ± 12.087
31–40 years	23 (11.5)	
41–50 years	70 (35.0)	
51–60 years	55 (27.5)	
61–70 years	31 (15.5)	
Over 70 years	16 (8.0)	
Gender		
Female	177 (88.5)	
Male	23 (11.5)	
Education		
Illiterate	6 (3.0)	
Less than High School Diploma	56 (28.0)	
High School Diploma	70 (35.0)	
Associate Degree	10 (5.0)	
Bachelor's Degree	46 (23.0)	
Master's Degree	10 (5.0)	
PhD	2 (1.0)	
Occupation		
Homemaker	133 (66.5)	
Retired	19 (9.5)	
Unemployed	3 (1.5)	
Employed	43 (21.5)	
Student	2 (1.0)	
Marital Status		
Single	13 (6.5)	
Married	147 (73.5)	
Divorced	15 (7.5)	
Widowed	25 (12.5)	
Number of Children		
0	28 (14.0)	2.070 ± 1.448
1	34 (17.0)	
2	84 (42.0)	
3	27 (13.5)	
4	13 (6.5)	
5	7 (3.5)	
6	5 (2.5)	
7	2 (1.0)	
Surgery		
No	52 (26.0)	
Yes	148 (74.0)	
Time since diagnosis (months)		49.909 ± 44.917
Time since treatment (months)		27.155 ± 31.722
Chemotherapy (sessions)		9.420 ± 8.876
Radiotherapy (sessions)		21.540 ± 12.906
Follow‐up duration (months)		37.071 ± 36.949

Abbreviations: M, mean; SD, standard deviation.

The initial results from the analysis of the components of PSQI indicated that 37.07 months after the last treatment, survivors took longer to fall asleep (95%), experienced more awakenings after sleep onset (88.5%), reported shorter sleep durations (72%), and had reduced sleep efficiency (86%) and impaired morning functioning (67%) in more than half of the survivors. These results are summarized in Table 2. It was also found that 86% experienced poor sleep quality, with difficulties in initiating and maintaining sleep being the most common complaints. Among the survivors, 6.5% reported severe sleep problems, 44.5% reported moderate issues, 47% reported mild sleep problems, and 2% reported no sleep issues based on the PSQI cutoff scores. The use of sleeping medications in the past month was reported by 28.5%. See Table 2 for Descriptive statistics of the variables.

**TABLE 2:** Descriptive statistics of questionnaire scores.

Variable	*M*	SD
Subjective sleep quality	1.295	0.844
Sleep onset latency	1.950	0.831
Sleep duration	1.610	1.215
Sleep efficiency	2.860	0.593
Sleep disturbances	1.675	0.694
Use of sleep medication	0.660	1.132
Morning dysfunction	1.135	0.996
Physical arousal	22.240	7.359
Cognitive arousal	18.850	7.221
Pre‐sleep arousal	41.090	12.937
Chronic pain level	21.950	17.400
General worry	34.150	2.898
Lack of worry	11.120	4.283
Worry	45.270	5.132
DBAS	34.740	7.740
Overall sleep quality	11.185	3.735

Abbreviations: DBAS, dysfunctional beliefs and attitudes about sleep; M, mean; SD, standard deviation.

The descriptive statistics of the study variables showed that the mean scores for pre‐sleep arousal, chronic pain levels, worry, DBAS, and sleep quality were 41.090, 21.950, 45.270, 34.740, and 11.185, respectively (see Table 2). For the analysis of the inferential statistics, correlation tests and multiple regression were employed (see Table 3).

**TABLE 3:** Correlation results among research variables.

Variable	Pearson's correlation coefficients, *r* (*p*)			
	Pre‐sleep arousal	Chronic pain level	Worry	DBAS
Chronic pain level	0.552 (0.000)	—		
Worry	0.161 (0.023)	0.141 (0.046)	—	
DBAS	0.363 (0.000)	0.294 (0.000)	0.032 (0.651)	—
Sleep quality	0.607 (0.000)	0.477 (0.000)	0.107 (0.132)	0.363 (0.000)

Abbreviation: DBAS, dysfunctional beliefs and attitudes about sleep.

The results of Pearson's correlation coefficient show a significant positive moderate relationship between pre‐sleep arousal and chronic pain (*r* = 0.552, *p* < 0.05), a significant positive weak relationship between worry (*r* = 0.161, *p* < 0.05), a significant positive moderate relationship between DBAS (*r* = 0.363, *p* < 0.05), and a significant positive strong relationship between poor sleep quality (*r* = 0.607, *p* < 0.05). A significant positive weak relationship was also observed between chronic pain and worry (*r* = 0.141, *p* < 0.05), a significant positive moderate relationship between DBAS (*r* = 0.294, *p* < 0.05), and a significant positive moderate relationship between poor sleep quality (*r* = 0.477, *p* < 0.05). Additionally, a significant positive correlation was observed between DBAS and poor sleep quality (*r* = 0.363, *p* < 0.05), indicating that higher scores in the variables under examination were associated with worse sleep quality. For a full list of correlations, see Table 3. For this purpose, in the next stage, hierarchical regression analysis was used to assess the predictive power of the independent variables on sleep quality.

**FIGURE 1:**
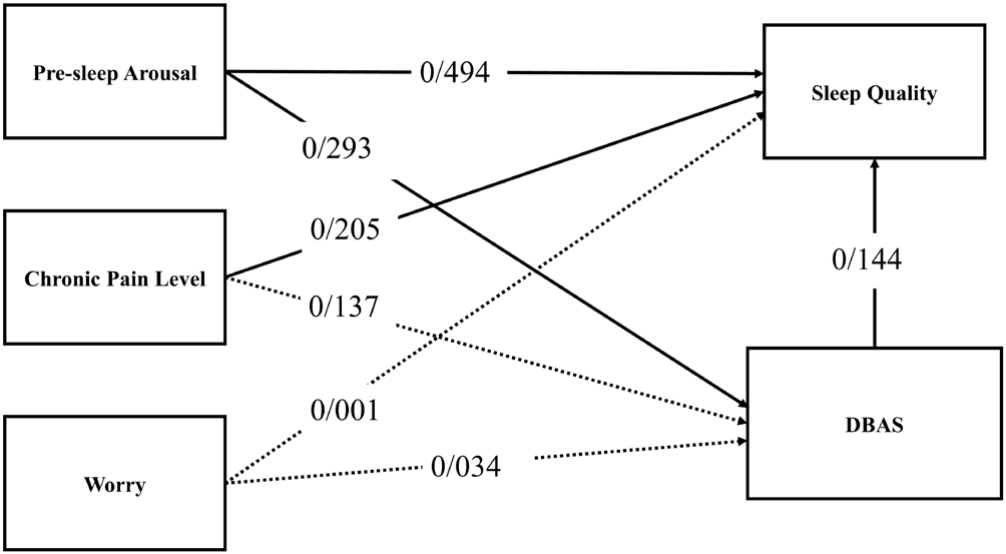
Mediation model of DBAS by independent variables (solid lines indicate significant pathways). DBAS, dysfunctional beliefs and attitudes about sleep.

Table 4 provides the coefficients and significance levels of these coefficients. In the first step, examining the regression coefficient for pre‐sleep arousal and the significance of the t‐value for this variable indicates that pre‐sleep arousal affects DBAS with a coefficient of 0.293. In the second step, examining the regression coefficients for pre‐sleep arousal and chronic pain and the significance of the t‐values for these variables indicates that they influence sleep quality with coefficients of 0.494 and 0.205, respectively. In the third step, examining the regression coefficients for pre‐sleep arousal, chronic pain, and DBAS, and the significance of their t‐values shows that these variables impact sleep quality with coefficients of 0.451, 0.185, and 0.144, respectively (see Table 4). Since the direct path coefficient between pre‐sleep arousal and DBAS, as well as the direct path between DBAS and sleep quality, is significant, the mediating role of DBAS between pre‐sleep arousal and sleep quality is confirmed. Moreover, regression analysis was used to assess the predictive power of sleep quality based on the components of pre‐sleep arousal, chronic pain, and worry, with the results as follows (Figure 1).

**TABLE 4:** Regression coefficients for predicting sleep quality based on pre‐sleep arousal, chronic pain, and worry, with DBAS as a mediator.

Step	Dependent variable	Independent variable	Standardized coefficients beta (*p*)	*T*
Step1	DBAS	Pre‐sleep arousal	0.293 (0.000)	3.677
		Chronic pain level	0.137 (0.086)	1.726
		Worry	−0.034 (0.610)	−0.510
Step2	Sleep quality	Pre‐sleep arousal	0.494 (0.000)	7.385
		Chronic pain level	0.205 (0.002)	3.076
		Worry	−0.001 (0.982)	−0.023
Step3	Sleep quality	Pre‐sleep arousal	0.451 (0.000)	6.614
		Chronic pain level	0.185 (0.006)	2.793
		Worry	0.004 (0.948)	0.065
		DBAS	0.144 (0.016)	2.437

Abbreviation: DBAS, dysfunctional beliefs and attitudes about sleep.

**FIGURE 2:**
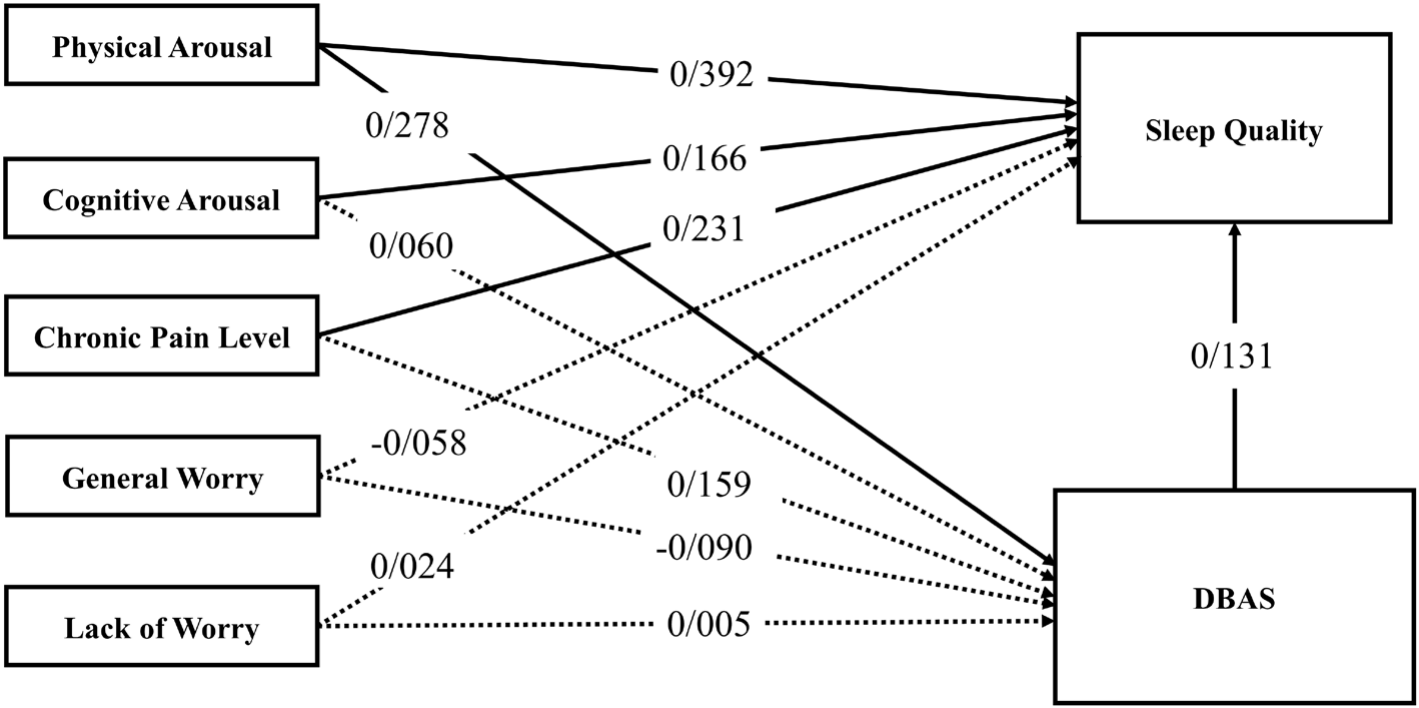
Mediation model of DBAS by pre‐sleep arousal, chronic pain, and worry components (solid lines indicate significant pathways). DBAS, dysfunctional beliefs and attitudes about sleep.

Table 5 provides the coefficients and their significance levels. In the first step, examining the regression coefficient for physical arousal and the significance of its t‐value indicates that this variable affects DBAS with a coefficient of 0.278. In the second step, examining the regression coefficients for physical arousal, cognitive arousal, and chronic pain and the significance of their t‐values indicates that these variables influence sleep quality with coefficients of 0.392, 0.166, and 0.231, respectively. In the third step, examining the regression coefficients for physical arousal, cognitive arousal, chronic pain, and DBAS, and the significance of their t‐values shows that these variables affect sleep quality with coefficients of 0.356, 0.158, 0.210, and 0.131, respectively. Since the direct path coefficient between physical arousal and DBAS, as well as the direct path between DBAS and sleep quality, is significant, the mediating role of DBAS between physical arousal and sleep quality is confirmed (see Table 5, Figure 2).

**TABLE 5:** Regression coefficients for predicting sleep quality based on physical and cognitive arousal, chronic pain, and worry, with DBAS as a mediator.

Step	Dependent variable	Independent variable	Standardized coefficients beta (*p*)	*T*
Step1	DBAS	Physical arousal	0.278 (0.001)	3.352
		Cognitive arousal	0.060 (0.519)	0.647
		Chronic pain level	0.159 (0.056)	1.922
		General worry	−0.090 (0.195)	−1.300
		Lack of worry	0.005 (0.937)	0.079
Step2	Sleep quality	Physical arousal	0.392 (0.000)	5.643
		Cognitive arousal	0.166 (0.034)	2.130
		Chronic pain level	0.231 (0.001)	3.322
		General worry	−0.058 (0.322)	−0.993
		Lack of worry	0.024 (0.669)	0.429
Step3	Sleep quality	Physical arousal	0.356 (0.000)	5.025
		Cognitive arousal	0.158 (0.042)	2.047
		Chronic pain level	0.210 (0.003)	3.022
		General worry	−0.046 (0.428)	−0.793
		Lack of worry	0.023 (0.675)	0.420
		DBAS	0.131 (0.029)	2.205

Abbreviation: DBAS, dysfunctional beliefs and attitudes about sleep.

## 4 Discussion

The main objective of our study was to examine the prevalence, severity, and psychological correlates of sleep quality in cancer survivors one to 5 years after treatment. The initial results from the analysis of the sleep quality questionnaire data indicated that more than half of the survivors had poor sleep quality. It was found that 37.07 months after the last treatment, survivors took longer to fall asleep, had more frequent awakenings after sleep onset, reported shorter sleep durations, and slept less than other individuals. It was also found that more than half of the participants experienced poor sleep quality, with difficulties in sleep onset and maintaining sleep being among the most common complaints. The high prevalence of insomnia was consistent with previous studies reported for long‐term cancer survivors (20%–90%) [29, 30, 31]. The use of sleeping medications in the past month was reported in our study, which was consistent with previous research on long‐term cancer survivors [29].

In the second part of the findings, it was also revealed that there was a significant positive correlation between pre‐sleep arousal, chronic pain, worry, DBAS, and poor sleep quality. As each of these factors increased, poor sleep quality also increased, and with their reduction, it can be expected that poor sleep quality would decrease. These results were consistent with multiple studies, including Chan et al. [5], Savard et al. [7], Lowery et al. [6], and Storo et al. [9]. Furthermore, given that the direct path coefficient between physical arousal and DBAS, as well as the direct path between DBAS and sleep quality, and the indirect path between pain and worry with DBAS are significant, the mediating role of DBAS between physical arousal, pain, worry, and sleep quality is confirmed.

According to Morin's integrated model [16], insomnia arises from the interaction between dysfunctional beliefs and cognitions, maladaptive habits and behaviors, worry about the consequences of sleep deprivation, and the development of issues such as fatigue, reduced performance, and mood decline, ultimately leading to arousal in its physiological, cognitive, and emotional dimensions. This model aligns with the results of our study. The findings of our study also revealed that cancer survivors who were frequently worried about cancer recurrence and metastasis experienced daytime worry and arousal, which led to psychological arousal before sleep [17]. This process was often accompanied by the onset of pain, resulting in the formation of DBAS and worry that insomnia would reoccur.

Most sleep studies among cancer survivors have focused on early survival (less than 3 years) or long‐term survival without time restrictions after treatment, emphasizing individual symptoms such as pain, fatigue, and depression, and their role in poor sleep outcomes [6, 8, 32]. However, this study focused on four different domains among cancer survivors, with pre‐sleep arousal showing the strongest association with sleep quality and sleep disturbances. In this context, Chan et al. (2023) identified two distinct trajectories of sleep disturbances among cancer survivors within the first 2 years post‐treatment [5]. Their study found that 30% of survivors experienced persistent sleep disturbances, while 70% showed symptoms of poor sleep or insomnia during the post‐treatment phase. These findings indicate that one out of every three cancer survivors continues to report sleep problems during the first 2 years after completing cancer treatment. Additionally, the overall prevalence of insomnia among survivors in their study is 54.8%, which is higher compared to the general population [33].

A key distinction of our study compared to previous research is its inclusion of individuals with various cancer types, whereas most prior studies have focused exclusively on women with specific cancers. According to previous studies [34, 35], sleep disturbances remain consistent over time. These findings suggest that, compared to issues like fatigue [35] and pain [36], which follow more varied trajectories after cancer treatment, improvements or reductions in sleep problems among patients occur less frequently. The observed improvement, deterioration, or persistence in sleep disturbance symptoms may reflect the contradictory nature of insomnia. For example, compensatory behaviors such as spending excessive time in bed while awake, preoccupation with sleep, and heightened focus on the negative consequences of sleep disturbances can exacerbate and perpetuate chronic sleep disorders.

Our study found that despite the significant time elapsed since their last treatment, cancer survivors continued to experience high levels of arousal, pain, moderate worry, DBAS, and poor sleep quality, as indicated by the assessment tool cutoff scores. These findings align with those reported by Dupont et al. [37]. They also concluded in their study that cancer survivors experienced intrusive thoughts before sleep, which significantly predicted sleep quality over the next 12 months. It has also been found that the constant recollection of the cancer experience interferes with sleep processes by increasing cognitive arousal [38]. Arousal strongly predicts sleep disturbance pathways and is more frequently experienced by individuals with severe sleep disorders. In this context, Chan et al. demonstrated that excessive arousal is recognized as the driving force behind chronic disturbed sleep and is associated with insomnia [5], which had previously been theorized as a hyperarousal disorder [39]. Thus, an increase in sleep disturbances naturally ensures the presence of hyperarousal, and hyperarousal also predicts the initial high level and gradual increase of sleep disturbances in breast cancer survivors [40].

Recent research shows that individuals with persistent sleep disturbances are more likely to report elevated levels of dysfunctional thoughts and attitudes, consistent with previous studies. One study assessing mood changes within 4 months of a cancer diagnosis found that these early symptoms were strongly linked to chronic sleep disturbances [35]. Another study found that higher levels of negative thoughts and beliefs, along with low mood, are naturally associated with poorer sleep, and these two variables often co‐occur [41]. Although there are limited studies on long‐term survivors after treatment regarding worry and pain, the existing evidence indicates that worry and rumination contribute to sleep problems in treated individuals [18].

Higher levels of pain have also been reported in women with poorer sleep quality, findings that are consistent with previous studies in cancer survivors [36, 42] and other populations [43]. According to Morin's integrated insomnia model [16], cancer survivors are often concerned about disease recurrence and metastasis, and many of them experience issues such as hot flashes, night sweats, sleep problems like insomnia, weight gain, joint stiffness, as well as worry, stress, anxiety, and various side effects during the post‐treatment period [17]. According to this model, hyperarousal plays a mediating role in insomnia, and since arousal regulates the balance between sleep and wakefulness, high levels of arousal are incompatible with sleep and can manifest in verbal (emotional‐cognitive), behavioral, and physiological (autonomic and central nervous system) ways. This model suggests that a set of triggering conditions can push arousal beyond its threshold and disrupt the sequence of relaxation, making it difficult to initiate sleep. These triggering conditions may also include external adverse events that occur during the day and manifest as sleep deprivation.

### 4.1 Limitations

The findings of our study indicate a high prevalence of sleep problems and poor sleep quality among cancer survivors. Factors such as worry, pain, DBAS, and pre‐sleep arousal were identified as correlated factors that play a significant role in the persistence of sleep problems in this group. Despite the aforementioned findings, this study also has several limitations that should be considered when generalizing the results. Firstly, the population of this study consisted of cancer survivors, and caution should be exercised when generalizing the findings to patients currently undergoing treatment. Secondly, the participants in this study had, on average, completed their last course of treatment 3 years prior, so caution is warranted when generalizing these findings to individuals who have recently concluded their treatment. Another limitation of our study is its correlational design, which precludes drawing causal inferences. Additionally, given its cross‐sectional nature, future research should adopt a longitudinal approach to track cancer survivors over a longer period and explore sleep disturbances in relation to other psychological factors such as rumination, emotion regulation, and psychological flexibility. Future studies should build on these findings to design and develop targeted interventions addressing the psychological factors associated with insomnia. Additionally, the lack of data on pre‐cancer sleep quality, detailed pain analysis, and specific measures about the fear of metastases represents limitations in our study. We acknowledge that not all causes of insomnia can be directly attributed to cancer, and we need to explore a wider range of potential causes in future studies. While we acknowledge the potential value of information about types of cancer, at this stage of research, we did not have access to this level of information. We attempted to collect this information but were limited due to ethical concerns. We acknowledge that this limits the interpretation of our results and that future research should examine the relationship between cancer type, the course of treatment, and sleep disturbances.

## 5 Conclusions

This study underscores the critical need to address sleep disturbances in cancer survivors, highlighting the significant impact of pre‐sleep arousal, pain, worry, and dysfunctional sleep beliefs on sleep quality. Persistent sleep problems after cancer treatment call for targeted interventions in survivorship care, including cognitive behavioral therapy for insomnia. Implementing sleep‐focused and cognitive behavioral strategies may significantly improve sleep quality and reduce the burden of insomnia‐related issues, enhancing overall well‐being in this vulnerable population.

## Author Contributions

All authors contributed to the study conception and design. Material preparation, data collection, and analysis were performed by Omid Amani, Mohammad Ali Mazaheri, Mona Malekzadeh Moghani, and Fariba Zarani. The first draft of the manuscript was written by Omid Amani, and all authors commented on previous versions of the manuscript. All authors read and approved the final manuscript.

## Ethics Statement

The Medical Ethics Committee of Shahid Beheshti University of Medical Sciences, Tehran, Iran (Ethical approval code: IR.SBMU.CRC.REC.1402.001) approved the study protocol.

## Consent

Informed consent was obtained from all individual participants included in the study. Publishing was agreed upon by all authors.

## Conflicts of Interest

The authors declare no conflicts of interest.

## Supporting information


Data S1.

## Data Availability

The data that support the findings of this study are available from the corresponding author upon reasonable request.
