# Supplementary material for: Prediction of Sleep Quality in Cancer Survivors Based on Arousal, Pain, and Worry: The Mediating Role of Dysfunctional Beliefs and Attitudes About Sleep
Source: Cancer Med. 2025 Apr 1;14(7):e70773. doi: 10.1002/cam4.70773 (PMC11959300; doi:10.1002/cam4.70773)
Supplement: Supplementary file 1 — Data S1. [file CAM4-14-e70773-s001.docx]

S1. Detailed Information on Assessment Measures

Pittsburgh Sleep Quality Index (PSQI):

Description: A self-report questionnaire assessing sleep quality over the past four weeks. Consists of 18 questions forming seven components: subjective sleep quality, sleep latency, sleep duration, sleep efficiency, sleep disturbances, daytime dysfunction, and use of sleep medication.

Scoring: Each component is scored from 0 to 3, with a global score ranging from 0 to 21. A score of 5 or higher indicates poor sleep quality.

Reliability: Buysse et al. (1989) reported a Cronbach's alpha of 0.83 and a test-retest reliability of 0.85 [1]. In Iran, Farahi et al. (2008) reported a Cronbach's alpha of 0.89 [2].

Dysfunctional Beliefs and Attitudes about Sleep Scale-10 (DBAS-10):

Description: A 10-item scale assessing dysfunctional beliefs and attitudes about sleep. Items are rated on a 5-point Likert scale.

Scoring: Scores range from 10 to 50.

Reliability: Validation studies have shown good reliability, with Cronbach's alpha of 0.77 for clinical populations and 0.79 for general populations [3]. An Iranian study reported a Cronbach's alpha of 0.84 [4].

Chronic Pain Grade (CPG):

Description: A questionnaire measuring the intensity of chronic pain across three dimensions: pain intensity, disability, and disability levels. Includes 7 items rated on an 11-point scale (0-10).

Scoring: Provides scores across three subscales: pain intensity, disability, and disability levels.

Reliability: Internal consistency of 0.91, with items scoring >0.75. Cronbach's alpha of 0.87 and 0.68 for disability and pain intensity subscales, respectively [30]. In Iran, Soleimani et al. (2019) reported a Cronbach's alpha of 0.89, and test-retest reliability of 0.89 using Spearman's correlation [5]. Tehrani et al. found Cronbach's alpha of 0.91 for disability and 0.83 for pain intensity [6].

Pennsylvania Worry Questionnaire (PSWQ):

Description: A 16-item self-report tool assessing worry. Items are scored on a 5-point Likert scale.

Scoring: Total scores range from 16 to 80.

Reliability: Validated in Iran by Barjalee et al. (2010), yielding a Cronbach's alpha of 0.88 [3].

Pre-sleep Arousal Scale (PSAS):

Description: A 16-item scale measuring pre-sleep arousal, divided into physical and cognitive arousal. Items are rated on a 5-point Likert scale.

Scoring: Total scores range from 16 to 80.

Reliability: Reliably differentiates between individuals with insomnia and healthy controls [7]. Shahzadi and Ejaz (2014) reported a Cronbach’s alpha of 0.87 and a test-retest reliability of 0.89 [8]. Hunstad et al. (2013) reported an internal consistency of 0.92 [9]. In Iran, Doos Ali Vand et al. (2014) reported an internal consistency of 0.85 and a test-retest reliability of 0.88 [10].

References

[1] Buysse DJ, Reynolds CF, 3rd, Monk TH, Berman SR, Kupfer DJ. The Pittsburgh Sleep Quality Index: a new instrument for psychiatric practice and research. Psychiatry Res. 1989;28(2):193-213. <https://doi.org/10.1016/0165-1781(89)90047-4>.

[2] Moghaddam JF. Validity and Reliability of the Persian version of the Pittsburgh Sleep Quality Index (PSQI) and PSI addendum: Kerman university of Medical science; 2009.

[3] Morin CM, Vallières A, Ivers H. Dysfunctional beliefs and attitudes about sleep (DBAS): validation of a brief version (DBAS-16). Sleep. 2007;30(11):1547-54.

[4] Vand HDA, Gharraee B, Farid AA, Rezvanifar S. Psychometric properties of short form Persian version of the Dysfunctional Beliefs and Attitudes about Sleep Scale. Iranian Journal of Psychiatry and Clinical Psychology. 2014.

[5] Soleymani A, Arani AM, Raeissadat SA, Davazdahemami MH. Validity and Reliability of the Persian Version of the Chronic Pain Grade Questionnaire in Patients with Musculoskeletal Pain. Advances in Nursing & Midwifery. 2019;28(3).

[6] Mirdrikvand F, Shirazitehrani A, Sepahvandi MA. The structural model of relationships between pain vulnerability, catastrophizing, fear and pain adjustment in patients with chronic musculoskeletal pain. Journal of Isfahan Medical School. 2017;34(413):1595-606.

[7] Nicassio PM, Mendlowitz DR, Fussell JJ, Petras L. The phenomenology of the pre-sleep state: the development of the pre-sleep arousal scale. Behav Res Ther. 1985;23(3):263-71. <https://doi.org/10.1016/0005-7967(85)90004-x>.

[8] Shahzadi N, Ijaz T. Reliability and validity of pre-sleep arousal scale for Pakistani University students. FWU Journal of Social Sciences. 2014;8(1):78.

[9] Hantsoo L, Khou CS, White CN, Ong JC. Gender and cognitive–emotional factors as predictors of pre-sleep arousal and trait hyperarousal in insomnia. Journal of psychosomatic research. 2013;74(4):283-9.

[10] Vand HDA, Gharraee B, Farid A-AA, Bandi MG. Prediction of insomnia severity based on cognitive, metacognitive, and emotional variables in college students. Explore. 2014;10(4):233-40.
